# ZIKV-Related Ideations and Modern Contraceptive Use: Cross-Sectional Evidence from the Dominican Republic, El Salvador, Honduras, and Guatemala

**DOI:** 10.4269/ajtmh.21-0765

**Published:** 2021-11-08

**Authors:** Julia M. Fleckman, Martha Silva, Jeni Stolow, Kendra LeSar, Kathryn Spielman, Paul Hutchinson

**Affiliations:** ^1^ Department of Social, Behavioral, and Population Sciences, Tulane University School of Public Health and Tropical Medicine, New Orleans, Louisiana;; ^2^ Department of International Health and Sustainable Development, Tulane University School of Public Health and Tropical Medicine, New Orleans, Louisiana;; ^3^ College of Public Health, Temple University, Philadelphia, Pennsylvania;; ^4^ Louisiana Department of Health, Office of Public Health, Baton Rouge, Louisiana;; ^5^ The Population Council, Washington, District of Columbia

## Abstract

Zika virus (ZIKV) can be sexually transmitted and can lead to severe neonatal and child health issues. The current study examines whether ZIKV-related ideational factors, including awareness of ZIKV and associated birth defects, are related to modern contraceptive use among women and men with sexual partners in four Latin American and Caribbean (LAC) countries. Data used are from cross-sectional household surveys conducted in 2018 in the Dominican Republic, El Salvador, Guatemala, and Honduras with representative samples of men and women aged 18–49 (*N* = 1,100). The association between self-reported use of modern contraception and measures of Zika knowledge, risk perceptions and social norms, and contraceptive self-efficacy was examined via sex disaggregated multivariate logistic regression models. Both men (OR 3.70, 95% CI 1.36–10.06, *P* < 0.05) and women (OR 3.71, 95% CI 2.30–5.99, *P* < 0.0001), who reported discussing family planning with their partner in the last year were more likely to use modern contraception compared with those who did not. Contrary to our hypothesis, knowledge that ZIKV can affect a fetus was negatively associated with modern contraceptive use for women (OR 0.49, 95% CI 0.29–0.85, *P* < 0.05). Given the cross-sectional nature of the survey, women not using contraception may be more likely to remember that ZIKV can affect a fetus. In the event of a related outbreak, future health promotion and communication efforts in LAC should focus on known determinants of modern contraceptive use, such as knowledge and partner communication, and knowledge of the health effects of ZIKV if pregnant, to influence family planning decision-making behavior.

## INTRODUCTION

In 2015 Zika virus (ZIKV) became the newest emerging infectious disease to infiltrate Latin America. To date, 86 countries and territories, mostly concentrated in Latin America and the Caribbean (LAC), have reported confirmed cases of ZIKV since the initial outbreak in 2015.^1^ ZIKV is a vector-borne virus in the genus *Flavivirus,* which can be transmitted via *Aedes* mosquito, blood transfusion, mother to child in utero, or by sexual intercourse. ZIKV, if acquired during pregnancy, can have adverse impacts on neonatal and child health.^1^^,^^2^ Since 2015 there have been over 600,000 confirmed cases of congenital ZIKV syndrome (CZS) across LAC.^3^ CZS is a distinct pattern of birth defects associated with ZIKV infection that can be present at birth or develop years after birth.^4^ The most common associated outcome of CZS has been microcephaly, which is related to poor brain growth, developmental disabilities, and death.^4^ CZS includes several other negative birth outcomes such as blindness, deafness, muscular dysfunction, hypertonia, seizures, and other structural brainstem issues.

The initial surge of CZS prompted several LAC ministries of health to issue recommendations for women of reproductive age to postpone or avoid pregnancy by using family planning until the ZIKV epidemic subsided to avoid exposure to ZIKV and CZS.^5^^,^^6^ This recommendation, although important in the context of the ZIKV epidemic, was issued in an environment where a large proportion (between one-third and a half) of pregnancies in the region are unintended.^7^^,^^8^ Recent estimates show that LAC encompasses one of the highest unintended pregnancy rates in the world at 96 per 1000 women aged 15–44 years.^9^ These rates, while still high, have been on the decline, partly due to the rise in family planning across the LAC over the last several decades. Among women of reproductive age (ages 15 to 49 years old) in these countries, prevalence estimates for modern contraceptive use include 55% in the Dominican Republic (DR), 49% in El Salvador, 31% in Guatemala, and 46% in Honduras.^10^

Even without Zika, the uptake of family planning in LAC is influenced by significant structural and social barriers that limit access and use of modern contraception. Young, low socioeconomic status, and rural populations especially experience lack of access to sexual and reproductive health services, resulting from fractured and inequitable health care systems, lack of insurance, unaffordable contraceptive methods, and restrictions on providers’ ability to provide evidence-based contraceptive information.^11^^–^^13^ The areas of highest ZIKV incidence were the same areas of impoverishment, violence, poor health care infrastructure, and lack of resources.^14^^,^^15^ Women in these areas were doubly imperiled by increased risk of ZIKV due to elevated exposure to mosquitos and increased risk of unplanned pregnancy due to limited health care access and resources,^16^^,^^17^ including irregular availability and/or low supply of contraceptives.^18^^,^^19^ Demand-side factors, such as pro-natalist religious ideologies, unequal gender roles, prohibitive social norms, and expectations regarding gender and sexuality, also limit the uptake of modern contraception in these countries.^20^^–24^ Gender inequalities contribute to the lack of dialogue among couples about family planning, and both men and women ranked the feasibility of using family planning methods including condoms as low, despite expressing a clear desire to protect unborn or future children from ZIKV.^23^

To explain demand-side factors affecting contraceptive uptake, the ideational model of communication and behavior change focuses on multilevel determinants of behaviors.^25^^,^^26^ Cognitive ideational factors such as knowledge, attitudes, beliefs, risk perceptions, and subjective norms; emotional ideations such as self-efficacy; and social ideations such as interpersonal communication, all potentially contribute to the likelihood of an individual practicing family planning.^25^ In the context of Zika, if women believe that they are highly susceptible to contracting the disease, that contraception (e.g., condom use) is effective in protecting against the disease, and that the disease is severe and would lead to harmful outcomes (such as microcephaly), they are more likely to use modern contraception to reduce this risk. They are also more likely to use modern contraception if the use is easy and they have confidence in their ability to practice the behavior, if they perceive that others are also using modern contraception, and if they can effectively communicate with their partners about contraception.^27^^,^^28^ The ideational model has been used to successfully examine and develop health communication campaigns to address HIV,^29^ malaria,^30^ pandemic influenza,^31^ pneumonia,^32^ and even contraceptive use.^33^^,^^34^

The current study utilizes the ideational model of communication and behavior change to investigate whether family planning and ZIKV-related ideational factors are related to the use of modern contraceptive methods among women and men with sexual partners in the DR, El Salvador, Guatemala, and Honduras. Cognitive ideational factors include those related to family planning (knowledge of modern contraceptives) as well as those related to ZIKV (knowledge, risk perceptions, subjective norms related to ZIKV, and ZIKV preventive behaviors). Emotional ideational factors related to family planning include self-efficacy related to partner communication and contraceptive use; and social ideation includes communication about family planning with partner. It is hypothesized that ZIKV-related factors specifically, including knowledge of ZIKV, knowledge of its associated birth defects when acquired during pregnancy, and risk perceptions for contracting ZIKV, will be associated with use of modern contraceptives.

## MATERIALS AND METHODS

### Study sample.

The analysis uses data from a representative sample of 2,600 men and women aged 18–49 years living in select US Agency for International Development (USAID)–funded project areas within the DR (*N* = 651), El Salvador (*N* = 672), Guatemala (*N* = 668), and Honduras (*N* = 609). Data were collected as part of a formative research study to inform the USAID ZIKV response, which examined knowledge, attitudes, and practices relating to ZIKV prevention behaviors, barriers, and motivators to the adoption of prevention behaviors. Information was also collected about exposure to ZIKV information through different channels.

A sampling frame of communities was developed based on ZIKV programming areas from USAID and other development partners at the time of the survey. A two-stage sampling approach was used.^35^ During the first stage, clusters were selected based on a probability proportional to populational size approach; larger clusters had higher probabilities of selection. During the second stage, a fixed target sample of households were selected in each cluster; thus, households in smaller clusters had a higher probability of selection. The sampling interval within each cluster was calculated based on a count of the buildings and houses in the randomly selected grid and random starting points were generated within the grids for the systematic sample of households. Individuals were randomly selected from a household roster of people who met the selection criteria. This approach ensured that everyone in the sampling frame had the same probability of being sampled. In households with more than one eligible man or woman, one respondent was randomly selected. Eligibility criteria included: 1) must be a resident in the household, and 2) must be between the ages of 18 and 49 years old.

The target sample size per country was 600, calculated to be able to obtain point estimates of key indicators with a margin of error of ±6% points. Response rates varied widely per country, from 44.6% in El Salvador, 56% in Honduras, 58.6% in Dominican Republic, and 81.3% in Guatemala. In this study, questions related to use of contraception were restricted to only those respondents who reported having a current partner, whether regular or casual. Therefore, for the current analysis, the initial sample of 2,600 was restricted to respondents who reported having at least one current partner (regular or casual), and who was not currently pregnant or did not have a pregnant partner (*N* = 1,457). The sample was then further restricted to those with no missing values for all variables of interest (*N* = 1,100). When comparing those omitted from this analysis to those included, those omitted were more likely to be older and have fewer years of education. Therefore, Little’s test of missing completely at random^36^ using the *mcartest* command in Stata/SE 16.0^37^ was used to assess if data were missing at random or if further data imputation was necessary. Data were found to be missing at random. Data used for the current study were available through a data use agreement with Breakthrough RESEARCH project and will be publicly available in July 2022.

### Survey instruments.

Selected respondents were invited to answer a survey collecting information on individuals’ sociodemographic information; knowledge of ZIKV transmission routes; ZIKV health effects; prevention methods for ZIKV; disease risk perceptions; knowledge, access to, and use of modern contraceptive methods; partner communication regarding family planning; perceived effectiveness and feasibility of prevention behaviors for ZIKV; self-reported mosquito prevention practices; and the observation of the use of lids for water storage containers. The questionnaire was initially piloted in Guatemala City as part of the field team training. Survey questions were assessed for comprehension with a sample of 20 participants. Following this pilot, the remaining three countries pretested the survey instrument to adjust for differences in local expressions as part of country-level field training.

### Ethical approval.

Ethical approval for this study was granted by respective local institutional review board in each country (Universidad Iberoamericana in the DR [CEI 2018-10], the Honduran Institute for Social Security [030-CB-HE], the El Salvador Ministry of Health [CNEIS/2018/011], and IRB Zugueme in Guatemala [PROZU/INVZU]) as well as the Tulane University Institutional Review Board (2018-652). Written informed consent to participate in the study was sought and obtained from all participants prior to commencing the survey.

### Measures.

#### Outcome variable.

The current use of modern contraception was measured for those who indicated they were using any of the following methods: female sterilization, male sterilization, intrauterine device (IUD), injectable, implant, pill, male condom, female condom, and other modern methods. Respondents who indicated use of at least one method were considered as currently using modern contraception, and those that indicated no use of any of the listed methods were considered as not currently using modern contraception.

#### Independent variables.

##### Family planning related ideation.

Discussion of family planning with partner in the last year was assessed with one binary item: “In the past year have you discussed family planning with your partner?” Participants responded with a “yes” or “no.” Perceived ease of starting a conversation with a partner about family planning was assessed with the item, “How easy or difficult is it to start a conversation about contraception with your partner? Is it very easy, easy, hard, or very hard?” Respondents who indicated it was very easy or easy were considered those who perceived partner communication as “easy,” and respondents who indicated it was very hard or hard were considered those who perceived partner communication as “hard.” Perceived ease of using condoms was assessed with a similar question specifically about condom use.

Knowledge of at least three modern family planning methods was assessed by asking participants who had received family planning information in the past year on which method(s) they had received information. Respondents who indicated knowledge of at least three forms of modern contraceptive were considered knowledgeable, and respondents who did not indicate knowledge of at least three forms modern contraceptive were considered unaware of modern family planning methods.

##### ZIKV-related ideation.

ZIKV risk perceptions were measured using questions regarding perceived risk of contracting ZIKV, and concern about contracting ZIKV. Regarding the risk of contracting ZIKV, respondents who indicated they had a high or medium chance of getting infected with ZIKV were considered as perceiving high risk, and respondents who indicated they had a low or no chance at all of getting infected with ZIKV were considered as perceiving low risk. Concern about ZIKV infection was assessed by asking how concerned participants would be if they became infected with ZIKV. Participants indicated if they would be very concerned, mildly concerned, or not very concerned. This was recoded into a binary variable with very concerned versus mildly or not very concerned.

Perceived descriptive norms of preventive behavior for ZIKV in the community were measured with a 5-point Likert scale by asking, “Please tell me whether you strongly agree, agree, neither agree nor disagree, disagree, or strongly disagree: Most people in my community do something to prevent ZIKV.” This was then recoded into a binary variable that combined strongly agree and agree as versus all other responses as “0”.

To assess overall knowledge of modes of transmission, we combined two questions: a question assessing knowledge of sexual transmission and a question assessing knowledge of vertical transmission. Those who had knowledge of modes of transmission included respondents who knew about sexual transmission or vertical transmission of ZIKV (unprompted) and those who did not have knowledge did not mention either mode of transmission. For knowledge of birth defects, those who had knowledge were those who mentioned birth defects as a potential outcome of ZIKV (unprompted) and those who did not have knowledge were those who did not. For either variables, respondents who reported never hearing of ZIKV were coded as unaware of sexual transmission, vertical transmission, or birth defects as an associated ZIKV health consequence.

##### Covariates.

Several covariates were assessed based on the ideational model of communication and behavior change that are commonly associated with differences in modern contraceptive use. Country (Guatemala, Honduras, El Salvador, and DR) and sex of the respondent (male/female) were recorded by the interviewer. Age was captured with four categories: 18–23, 24–30, 31–39, and 40–49. Education was captured with three categories: primary school or less, incomplete secondary, complete secondary, and more than secondary. A wealth index for each country was generated based on responses to questions about household living conditions, such as source of water, type of toilet, and dwelling with finished walls or floor. The index also included indicators of ownership of household consumer durables, such as a mobile phone, refrigerator, television, radio, sofa, washing machine, computer, kitchen cabinet, car, motorcycle, or bicycle.^38^ The principal components analysis command *pca* was performed on these indicators in Stata 16.0, and a score variable was created based on the eigenvalues for the first principal component. Households were than ranked and divided into quintiles based on their score on the index.^39^

### Data analysis.

Data for this study were pooled across countries. Sex-disaggregated univariate, bivariate, and multivariate analyses were conducted. Analyses were weighted to account for sampling probability and to represent the distribution of men and women in census areas. For each country, sampling weights consisted of the inverse of probability of selection at stage 1 multiplied by the probability of selection at stage 2. The data were then pooled and reweighted to normalize the weight variability by country. All analyses were conducted using Stata/SE 16.0.

Descriptive, univariate analyses included frequency distributions for all variables of interest. χ^2^ analyses were used to test for bivariate associations between use of modern contraception and relevant ideational factors, such as knowledge of ZIKV and family planning methods, self-efficacy in partner communication regarding family planning, perceived risk of contracting ZIKV while pregnant, and perceived subjective social norms related to ZIKV. To define statistically significant associations, standard errors and *P* values were used.

Multivariate logistic regression models were used to investigate the effects of the independent variables of interest on self-reported use of modern contraception. Model 1 presents associations between family planning–related ideational variables and the use of modern contraceptive methods, controlling for covariates, while model 2 includes ZIKV-related ideational variables, controlling for family planning related ideations and covariates.

To test for issues of multicollinearity among independent variables, we used the post=estimation *collin* command in Stata to estimate variance inflation factors. In all models, we found that estimated variance inflation factors did not exceed three, supporting the hypothesis that interdependence amongst model covariates was not problematic in affecting regression coefficients.^40^

## RESULTS

Demographic characteristics are presented in Table 1. Differences in the use of modern contraception methods were examined in 1,100 adults with partners. The sample consisted of respondents from four Spanish-speaking countries in LAC (DR = 26%, El Salvador = 20%, Honduras = 25%, Guatemala = 29%), 63% were female, and the majority of respondents were 31–39 (29%) or 40–49 (36%) years of age. Most participants had an incomplete or complete secondary (43%) or more than secondary (19%) education and wealth quintiles were evenly distributed. The majority of men had at least some secondary education (99%), but most women reported having a secondary education or less (78%). Most male (58%) and female (70%) respondents were between the ages of 31 and 49 years old. The majority of male respondents reported they were in the richest or upper middle quintiles for wealth (59%), whereas female respondents were more likely to report being in the poorest, lower middle, or middle wealth quintiles.

**Table 1:** Weighted demographic characteristics of sample (*N* = 1,100)*

	Total	Men	Women
	% (*N* = 1,100)	% (*N* = 298)	% (*N* = 802)
Country			
DR	25.5	22.7	27.2
El Salvador	20.4	24.0	18.2
Honduras	25.0	20.6	27.6
Guatemala	29.2	32.7	27.1
Age			
18–23	11.6	14.2	10.1
24–30	23.2	28.1	20.3
31–39	28.7	26.1	30.2
40–49	36.5	31.6	39.4
Education			
Primary or less	37.2	31.3	40.7
Incomplete or complete secondary	43.4	53.2	37.6
More than secondary	19.4	15.5	21.7
Wealth quintiles			
Poorest	18.2	14.0	20.8
Lower Middle	18.1	13.6	20.8
Middle	22.2	14.7	26.6
Upper Middle	20.8	27.0	17.1
Richest	20.7	30.6	14.8

DR = Dominican Republic.

* Includes data from the Dominican Republic, El Salvador, Guatemala, and Honduras collected in 2018.

Frequency distributions including the outcome of interest, modern contraceptive use, and independent variables are also presented in Table 1. Approximately 55% of male respondents and 67% of female respondents indicated current use of a modern contraceptive method. Approximately 14% of men and 8% of women reported having the desire to become pregnant (themselves or their partners) in the next year. Over half (51%) of men and 48% reported having discussed family planning with partner. Most respondents reported they believed that it is easy to start a conversation with partner about family planning (91% for both men and women) and believed that it is easy to use condoms (64% among males and 56% among females). Only 30% of men and 31% of women reporting knowing of at least three forms of modern contraception.

ZIKV-related ideations were mixed with few respondents reporting they thought there is a high chance of becoming infected with ZIKV (30% among males and 26% among females), and most respondents reporting being very concerned about ZIKV infection (87% and 83%). About half of both male and female respondents agreed that most people in their community do something to prevent ZIKV (53% among males and 52% among females). Very few participants knew that ZIKV can be sexually or vertically transmitted (16% among males and 25% among females) and that it may affect a fetus (16% among males and 28% among females).

Bivariate analyses including the outcome of interest, modern contraceptive use, and independent variables are shown in Table 2. Men who reported social ideations (having discussed family planning with partner in the last year) (*P* < 0.01), and emotional ideations (belief that it is easy to use condoms) (*P* < 0.05) were more likely to report using modern contraception than those that do not report having discussed or ease of use. Among female respondents, those who reported social ideations (having discussed family planning with partner in the last year) (*P* < 0.0001), emotional ideations (belief that it is easy to start a conversation with partner about family planning) (*P* < 0.05), knowledge related to family planning (having heard of at least three modern contraception method in the past year) (*P* < 0.0001), and cognitive ideations related to ZIKV (belief that there is a high risk of becoming infected with ZIKV) (*P* < 0.01), were also more likely to report using modern contraception compared with those who did not have a discussion or report ease of communication or contraceptive use.

**Table 2:** Weighted proportions of respondent’s behavior, intention, ideation, and knowledge among men and women by use of modern contraception (*N* = 1,100)†

	Men (*N* = 298)			Women (*N* = 802)		
	Total %	No current use %	Current use %	Total %	No current use %	Current use %
***Behavior***						
Currently use a modern family planning method	54.9	–	–	66.5	–	–
**Family Planning related ideation**						
***Social Ideation***						
Discussed family planning with partner in last year	50.8	37.7**	61.5	47.6	30.7***	56.1
***Emotional Ideation***						
Very easy or easy to start a conversation with partner about family planning	90.5	88.9	91.9	91.1	86.6*	93.4
Very easy or easy to use condoms	63.5	56.9*	69.0	56.0	64.7	51.6
***Knowledge***						
Know of at least three modern contraceptive methods in the past year	30.1	24.2	35.0	30.5	21.5***	35.1
**Zika-related ideation**						
***Cognitive Ideation***						
Perceived high risk of getting ZIKV	29.5	32.1	27.3	25.8	32.0**	22.7
Very concerned about ZIKV infection	87.1	92.5	82.6	82.8	90.5	78.9
Strongly agree or agree that most people in the community do something to prevent ZIKV	52.8	49.9	55.2	54.4	52.0	55.7
***Knowledge***						
Know about ZIKV sexual and vertical transmission	16.4	19.0	14.3	24.9	15.0	29.8
Know that ZIKV may affect a fetus during pregnancy	16.0	20.6	12.2	27.5	28.2	27.1

ZIKV = Zika virus.

* *P* < 0.05, ** *P* < 0.01 *** *P* < 0.001.

†Includes data from the Dominican Republic, El Salvador, Guatemala, and Honduras collected in 2018.

Results from the multivariate model disaggregated by sex are shown in Table 3. Some family planning ideations were significantly associated with modern contraceptive use. Social ideations played a role in use of contraceptives for both men and women. Specifically, men (aOR 3.70, 95% CI 1.36–10.06, *P* < 0.05) and women (aOR 3.71, 95% CI 2.30–5.99, *P* < 0.0001) who reported discussing family planning with their partner in the last year were more likely to use modern contraception compared with those who did not. Knowledge of family planning mattered for women. Women who knew of at least three modern family planning methods were more likely to report use of a modern family planning method compared with those who reported not knowing of at least three methods (aOR 2.04, 95% CI 1.36–3.06, *P* < 0.0001). In terms of Zika-related ideations, women with knowledge that ZIKV can affect a fetus by causing birth defects were significantly less likely to use modern contraception compared with women without such knowledge (aOR 0.49, 95% CI 0.29–0.85, *P* < 0.05). Women who had knowledge of sexual or vertical transmission of ZIKV were more likely to use modern contraception (aOR 0.49, 95% CI 0.92–4.70, *P* < 0.10).

**Table 3:** Multivariate models of associations between ZIKV and family planning–related ideations and use of modern contraceptive methods

	Men (*N* = 298)	Women (*N* = 802)
	aOR	aOR
	(95% CI)	(95% CI)
**Family Planning–Related ideation**		
***Social Ideation***		
Discussed family planning with partner in last year	3.73	3.55
	(1.41**–**9.84)**	(2.22**–**5.69)***
***Emotional Ideation***		
Very easy or easy to start a conversation with partner about family planning	1.49	1.45
	(0.46**–**4.74)	(0.81**–**2.61)
Very easy or easy to use condoms	2.12	0.71
	(0.83**–**5.38)	(0.29**–**1.73)
***Knowledge***		
Know of at least three modern family planning method in the past year	1.78	1.99
	(0.52**–**6.14)	(1.34**–**2.97)***
**Zika related ideation**		
***Cognitive Ideation***		
Perceived high risk of getting ZIKV	0.72	1.14
	(0.28**–**1.85)	(0.62**–**2.07)
Very concerned about ZIKV infection	0.80	0.50
	(0.20**–**3.16)	(0.23**–**1.07)†
Strongly agree or agree that most people in the community do something to prevent ZIKV	1.10	0.89
	(0.47**–**2.57)	(0.52**–**1.52)
***Knowledge***		
Know about ZIKV sexual and vertical transmission	0.71	2.08
	(0.14**–**3.52)	(0.92**–**4.70)†
Know that ZIKV may affect a fetus during pregnancy	0.69	0.52
	(0.14**–**2.75)	(0.30**–**0.91)*

ZIKV = Zika virus.

* *P* < 0.05, ** *P* < 0.01, *** *P* < 0.001, † *P* < 0.1.

## DISCUSSION

The current study extends previous research on modern contraceptive use and the ZIKV outbreak by not only examining the association between modern contraceptive use and knowledge and self-efficacy related to use, but also ideational factors related to ZIKV including risk perceptions and subjective norms. A large portion of respondents (62%) reported current use of at least one type of modern family planning method. Numbers representing women’s use of modern contraceptives are higher than those of other similar studies.^18^^,^^41^ Later implementation of the study’s survey in 2018, a few years after the 2016 ZIKV outbreak peak, may explain the higher reported rate of contraceptive use. By this time, many organizations had already responded to the ZIKV outbreak via educational campaigns, provision of condoms, or health programming specific to increasing family planning.^22^ These responses may have yielded increased access for priority populations, which could account for this study’s high rate of contraceptive use as compared with earlier 2015–2017 knowledge, attitudes, and practices (KAP) studies in the region.^12^^,^^15^ Further, there has been an upward trend in contraceptive use in LAC for the past 25 years due to partnerships between NGOs and national governments working toward increased access to family planning services.^13^ These findings highlight the importance of efforts to increase utilization of modern contraceptives.

The only significant association for ZIKA-related ideations was an inverse relation between knowledge that ZIKV can affect a fetus by causing birth defects and contraceptive use for women only. This was contrary to our a priori hypothesis. Salience may play a particularly important role in explaining this association.^42^^–^^44^ Given that the data are cross-sectional, it may be possible that this association is driven by modern contraceptive use, where women who are not using contraceptives, possibly due to fertility intentions or structural and normative gender-based barriers,^20^^–^^22^^,^^45^ are more aware of their risk of becoming pregnant and thus more likely to remember the potential risks to a fetus when contracting ZIKV. However, further investigation is needed to understand the relationship between knowledge of birth defects and vertical transmission of ZIKV as they relate to contraceptive use for women, including access to contraceptives.

There was no significant relation for both men and women between ZIKV-related cognitive ideations including concern about contracting ZIKV, perceived risk of contracting ZIKV, community social norms regarding ZIKV, and modern contraceptive use. The lack of association is an important finding for addressing sexual and vertical transmission of ZIKV and other outbreaks. Null results indicate that risk perceptions and subjective norms regarding the disease and transmission may not be the most effective points for intervention to increase use of modern contraceptives as a preventive behavior. Instead, family planning ideations may be more useful to target for behavioral change.

The current study did identify an association between family planning social ideations, including partner communication, and modern contraceptive use for both men and women. Respondents who reported family planning conversations with their partners as very easy or easy were more likely to use a modern family planning method, compared with those who reported conversations about family planning with their partners to be difficult. Sexual and reproductive health education interventions that address partner communication skills have been effective in improving contraceptive use.^46^ Qualitative research conducted in the DR in the context of ZIKV also highlights the need for male involvement in reproductive health programming to promote joint decision-making about family planning, and condom use in particular.^24^ Although results from the current study leave some uncertainty about whether self-efficacy to communicate with a partner about contraceptives leads to greater use of contraceptives, or vice versa, improving partner communication may be a more effective intervention point for increasing more consistent contraceptive use in the context of an outbreak that includes partner transmission.

Additionally, the current study identified an association between knowledge of modern contraceptive methods and use of modern contraceptives for women, as respondents who reported having heard of at least three modern contraceptive methods in the past year were more likely to use modern contraceptives than those who had had heard of two or less. Increasing knowledge of contraceptive methods is a common intervention component for increasing use of modern contraceptives,^47^ and should not be overlooked in the context of an emergency response.

The current study has several limitations. First, it is cross-sectional and therefore, no conclusions about the sequence of events between exposure and outcome variables can be assessed. Further, there is also the potential for self-selection bias based on the household sampling design and low response rate in three out of four countries. Another threat to internal validity is information bias. Specifically, measurement bias could be an issue as exposure variables were based solely on self-report. Recall and social desirability biases may be an issue by asking about past behavior and family planning behaviors. Lastly, the low sample size for male respondents limits our power to detect statistically significant associations.

## CONCLUSIONS

Findings from this study are relevant for future programming in ZIKV programming and family planning and sexually transmitted disease prevention. In particular, the association between family planning ideations and contraceptive use indicate that knowledge of modern contraceptives and partner communication may be more important targets to promote contraceptive use. Although no longer an epidemic, ZIKV is still endemic throughout the LAC. Health promotion and communication targeting family planning are essential in the region as this is still an area of high unmet family planning need and endemic ZIKV. It is imperative that in the future public health emergencies that affect birth outcomes, such as ZIKV, and health promotion focus on the known determinants of contraception use, such as knowledge and partner communication, instead of relying on the fear of the outbreak to change fertility decision-making behavior.
